# Perturbances in Both Circulating B and CD4^+^ T Cells Discriminate Multiple Sclerosis from Other Central Nervous System Autoimmune Diseases

**DOI:** 10.1002/eji.70049

**Published:** 2025-09-03

**Authors:** Laurens Bogers, Jasper Rip, Suzanne C. Franken, Kirsten L. Kuiper, Ana M. Marques, Annet F. Wierenga‐Wolf, Marie‐José Melief, Cato E. A. Corsten, Romy A. M. Klein Kranenbarg, Janet de Beukelaar, Ide Smets, Beatrijs H. Wokke, Maarten J. Titulaer, Joost Smolders, Marvin M. van Luijn

**Affiliations:** ^1^ Department of Immunology MS Center ErasMS Erasmus University Medical Center Rotterdam The Netherlands; ^2^ Department of Neurology Erasmus University Medical Center Rotterdam The Netherlands; ^3^ Department of Neurology MS Center ErasMS Erasmus University Medical Center Rotterdam The Netherlands; ^4^ Department of Neurology Albert Schweitzer Hospital Dordrecht The Netherlands; ^5^ Neuroimmunology Research Group Netherlands Institute for Neuroscience Amsterdam The Netherlands

**Keywords:** anti‐AQP4 NMOSD, autoimmune encephalitis, disease progression, disease specificity, MOGAD

## Abstract

Using spectral flow cytometry, we analyzed circulating lymphocyte subsets in treatment‐naive individuals with multiple sclerosis (MS) and other central nervous system autoimmune diseases (CNS AIDs). Elevated B‐cell and CD4^+^ T‐cell frequencies were a disease‐specific feature of MS, while reduced T‐bet^+^ and CXCR3^+^ B‐cell levels were associated with progressive disease.

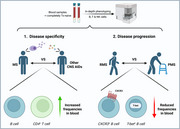

B cells play a central role in many central nervous system autoimmune diseases (CNS AIDs), including multiple sclerosis (MS) [1]. For the development of MS, B cells are likely misinformed by CD4^+^ T helper cells both outside and inside the CNS [2]. As a result, memory B cells enter the CNS and subsequently differentiate into antibody‐secreting cells (ASCs) [2, 3, 4]. Locally produced antibodies likely contribute to MS pathology, although a specific target antigen has not been identified [5]. Other CNS AIDs such as autoimmune encephalitis (AE) and rare demyelinating diseases (RDD), including AQP4‐IgG^+^ neuromyelitis optica spectrum disorder (NMOSD) and MOG antibody‐associated disease (MOGAD), are characterized by specific circulating CNS‐directed antibodies [6, 7]. For such diseases, intrathecal antibody production is less common, but highly variable, and thus cannot be seen as a specific immunological hallmark of MS [1]. Besides for differences in antibody specificity and compartmentalization, we do not know if the distribution and features of B cells amongst other circulating lymphocytes could be useful to discriminate MS from other CNS AIDs. A major hurdle is that steroid treatment often confounds the interpretation of such disease‐specific cellular alterations.

To address this, we performed spectral flow cytometry on blood samples from healthy individuals (young/old) and people with MS (relapsing/progressive), AE (anti‐LGI1/‐IgLON5/‐GAD65), or RDD (anti‐AQP4 NMOSD/MOGAD) who did not receive any immunomodulatory treatment at the time of sampling (Figure 1A).

**FIGURE 1:**
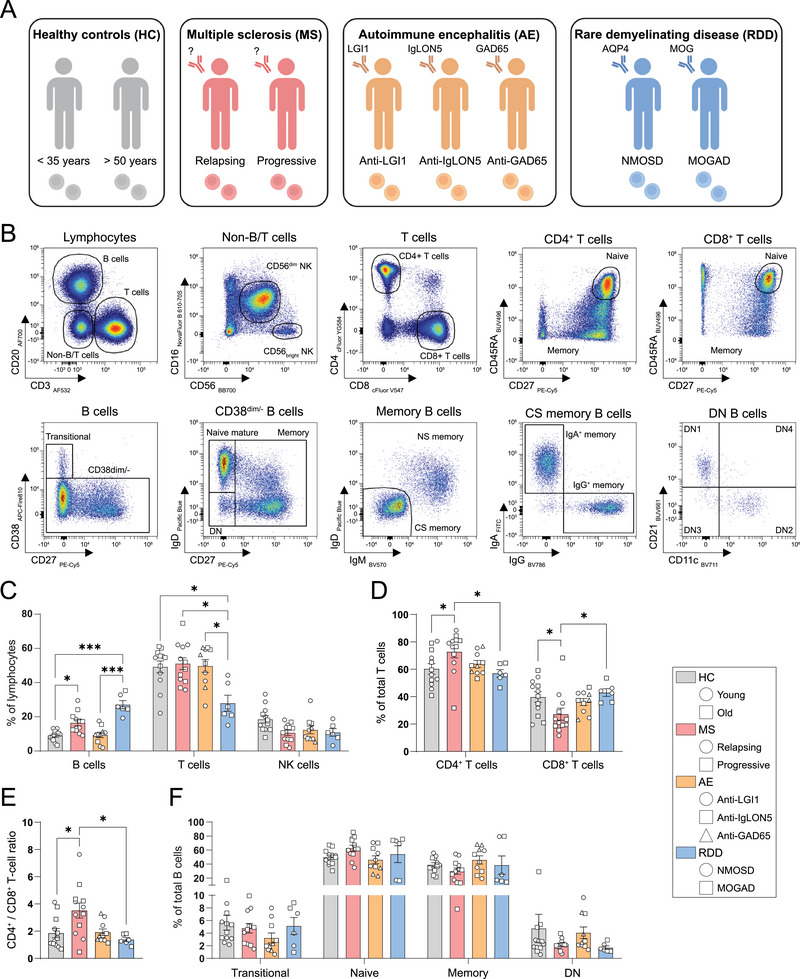
Increased B‐ and CD4^+^ T‐cell frequencies in blood of people with MS. (A) Thawed blood samples from healthy controls (young/old; *n* = 12) and treatment‐naive individuals with MS (relapsing/progressive; *n* = 12), AE (anti‐LGI1/‐IgLON5/‐GAD65; *n* = 10) or RDD (anti‐AQP4 NMOSD/MOGAD; *n* = 6) were analyzed using ex vivo spectral flow cytometry. (B) Gating strategy of B‐, T‐, and NK‐cell subsets. (C) B‐, T‐, and NK‐cell frequencies within viable lymphocytes. (D) CD4^+^ and CD8^+^ frequencies within total T cells. (E) CD4^+^/CD8^+^ T‐cell ratios. (F) Transitional, naive mature, memory, and double negative (DN; CD27^−^IgD^−^) frequencies within total B cells. All data are presented as mean ±SEM and analyzed using Kruskal‐Wallis and Dunn's post hoc tests. Outliers were identified and excluded using Grubbs’ test. *p < 0.05, ***p < 0.001.

First, we evaluated differences in major lymphocyte subsets within thawed blood samples (Figure 1B). While NK‐cell frequencies were similar between groups, T‐cell frequencies were reduced in RDD, and B‐cell frequencies were notably increased in both MS and RDD (Figure 1C). CD4^+^ T cells were significantly elevated, while CD8^+^ T cells were significantly diminished in MS, as reflected by frequencies (Figure 1D) and CD4^+^/CD8^+^ T‐cell ratios (Figure 1E). The observed effects were not sex‐dependent (Figure S1A,B). No major differences were observed in other B‐, T‐, and NK‐cell subpopulations (Figure 1F; Figure S1C–G). The selective enrichment of both circulating B cells and CD4^+^ T cells in people with MS points to disease‐specific changes in lymphocyte fractions and thereby could reflect a distinct underlying mechanism per antibody‐associated CNS AID group, which requires validation and further investigation in the near future.

To better understand the implications of elevated B‐ and CD4^+^ T‐cell frequencies in MS, we further analyzed whether T‐bet and CXCR3, as markers of CD4^+^ T‐cell interaction and CNS‐homing, are differentially expressed by B cells in the MS versus the RDD and AE groups. First, we observed no sex‐related effects on T‐bet and CXCR3 expression (Figure S2A,B). No major differences were found when comparing disease groups in total B cells and memory populations, although we did observe a trend toward lower T‐bet^+^ and CXCR3^+^ B‐cell frequencies in MS and RDD (Figure 2A,B; Figure S2C,D). Within the MS group, the proportions of both T‐bet^+^ and CXCR3^+^ B cells were markedly reduced in people with advanced progressive versus early relapsing disease (Figure 2A,B; Figure S2C,D), a pattern not observed for CD4^+^ memory T cells (Figure S3A,B). No reduction was found when comparing aged versus young healthy individuals (Figure 2A,B; Figure S2C,D), suggesting that age is not a determining factor for these lower proportions. Additionally, circulating memory B cells from the progressive MS subgroup showed decreased differentiation into CD27^high^CD38^high^ ASCs under in vitro conditions that mimic CD4^+^ T‐cell help, which was IFN‐γ‐dependent (Figure 2C). CXCR3 expression on in vitro‐induced ASCs positively correlated with that on ex vivo memory B cells (Figure 2D) and was lower in the advanced progressive than the early relapsing subgroup (Figure S4A,B). We validated our findings by using another MS cohort, in which ex vivo ASCs could be measured in fresh blood samples and showed a significant reduction in people with progressive versus relapsing disease (Figure 2E). These data may suggest that CXCR3^+^ memory B cells—which drive hyperreactive B–T cell interactions in MS^2^ and show a high ASC differentiation capacity [8]—could redistribute to the CNS of individuals with longstanding progressive MS [3, 4]. The compartmentalization of such B cells in the CNS associates with worse disease outcomes [9] and may explain the limited efficacy of B‐cell depletion therapies in chronic stages of MS [10], as anti‐CD20 monoclonal antibodies cannot reach the CNS.

**FIGURE 2:**
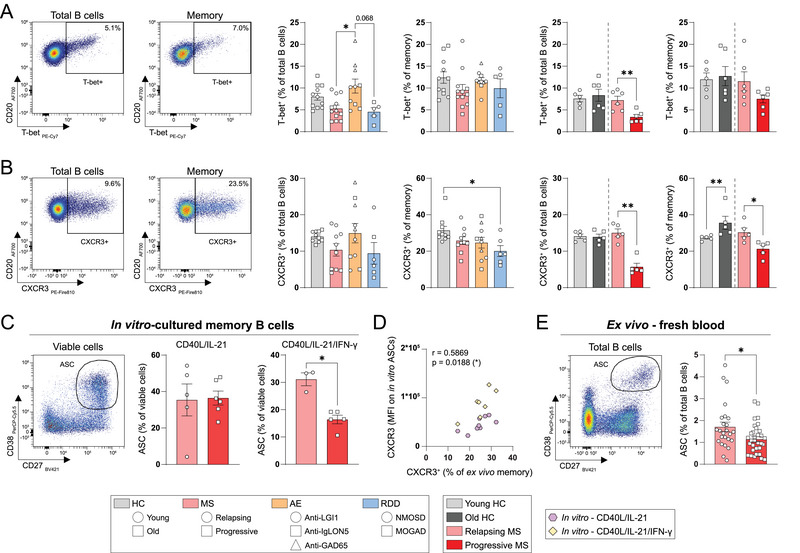
Reduced frequencies of T‐bet^+^ and CXCR3^+^ B cells in the blood of people with progressive MS. (A, B) Representative gating and frequencies of T‐bet^+^ and CXCR3^+^ populations within total and memory B cells. (C) Antibody‐secreting cell (ASC) frequencies from in vitro‐cultured memory B cells of treatment‐naive people with relapsing and progressive MS (*n* = 3–6). (D) Correlation plot showing the relation between ex vivo CXCR3 expression on memory B cells and in vitro CXCR3 expression on ASCs. (E) ASC frequencies within fresh blood samples from treatment‐naive people with relapsing (*n* = 25) and progressive (*n* = 33) MS. All data are presented as mean ±SEM and analyzed using Kruskal–Wallis and Dunn's post hoc tests (A, B), Mann–Whitney *U*‐tests (A, B, C, E) or Spearman correlation coefficients (D). Outliers were identified and excluded using Grubbs’ test. *p < 0.05, **p < 0.01.

Taken together, these findings highlight the increase of circulating B cells and CD4^+^ T cells as a specific characteristic of MS among CNS AIDs, for which a reduction in circulating T‐bet^+^ and CXCR3^+^ B cells is associated with progressive disease regardless of age.

## Author Contributions

**Laurens Bogers**: Conceptualization, data curation, formal analysis, investigation, methodology, project administration, validation, visualization, writing – original draft. **Jasper Rip**: Conceptualization, investigation, methodology, project administration, validation, writing – review and editing. **Suzanne C. Franken**: Resources, investigation, project administration. **Kirsten L. Kuiper, Ana M. Marques**: Investigation. **Annet F. Wierenga‐Wolf, Marie‐José Melief**: Investigation, methodology. **Cato E.A. Corsten, Romy A.M. Klein Kranenbarg, Janet de Beukelaar, Ide Smets, Beatrijs H. Wokke**: Resources. **Maarten J. Titulaer**: Conceptualization, funding acquisition, resources, project administration. **Joost Smolders**: Conceptualization, funding acquisition, resources, methodology, project administration, supervision, validation, writing – review and editing. **Marvin M. van Luijn**: Conceptualization, funding acquisition, methodology, project administration, supervision, validation, writing – review and editing.

## Ethics Statement

All donors provided written informed consent. Study protocols were approved by the medical ethics committee of the Erasmus Medical Center (2021‐0251; 2021‐0946; 2019‐0845).

## Conflicts of Interest

I.S. has received speaker fees from Biogen, Merck, and Sanofi. M.J.T. has filed a patent, on behalf of the Erasmus MC, for methods for typing neurological disorders and cancer, as well as devices for use therein, and has received research funding from EpilepsieNL (NEF 14‐19 & 19‐08), Dioraphte (2001 0403), ZonMw (Memorabel fellowship, VIMP scheme and Open Competition [ACT‐MD]), ItsME, Erasmus MC Foundation, and Erasmus Trustfonds. M.J.T. has served on the scientific advisory board of Horizon Therapeutics/AmGen, ArgenX, and Arialys; for consultation at Guidepoint Global LLC, and at UCB. M.J.T. is co‐PI of the EXTINGUISH trial. M.J.T. has received unrestricted research grants from Euroimmun AG and CSL Behring. M.J.T. has filed a copyright on behalf of ErasmusMC for the PROSE (Patient‐Reported Outcome Scale in Encephalitis). J.S. reports grants for scientific research from Biogen, Hansa Biopharma, Roche, and Siemens Healthineers, and has received speaker and/or consultancy fees from Biogen, Merck, Novartis, Roche, and Sanofi. M.M.v.L. has received research support from EMD Serono, Merck, Novartis, GSK, and Idorsia Pharmaceutical Ltd. The remaining authors declare no conflicts of interest.

## Peer Review

The peer review history for this article is available at https://publons.com/publon/10.1002/eji.70049.

## Supporting information




**Supporting file 1**: eji70049‐sup‐0001‐SuppMat.pdf

## Data Availability

The data that support the findings of this study are available from the corresponding author upon reasonable request.
