# Supplementary material for: Perturbances in Both Circulating B and CD4+ T Cells Discriminate Multiple Sclerosis from Other Central Nervous System Autoimmune Diseases
Source: Eur J Immunol. 2025 Sep 3;55(9):e70049. doi: 10.1002/eji.70049 (PMC12405970; doi:10.1002/eji.70049)
Supplement: Supplementary file 1 — Supporting file 1: eji70049‐sup‐0001‐SuppMat.pdf [file EJI-55-e70049-s001.pdf]

Supporting information

Methods

Patients

Thawed blood samples from healthy individuals (young and old; n=12), as well as people with MS (relapsing and progressive; n=12), AE (anti-LGI1, -IgLON5, and -GAD65; n=10), and RDD (anti-AQP4 NMOSD and MOGAD; n=6), were used for *ex vivo* phenotyping and/or *in vitro* differentiation assays. Fresh blood samples from relapsing (n=25) and progressive (n=33) MS donors were used for *ex vivo* phenotyping. Relapsing patients were defined as those with a relapsing onset and no progressive disability at the time of sampling, whereas progressive patients were defined as those with progressive disability at the time of sampling, regardless of whether their onset was relapsing or not. All individuals included in this study did not receive any kind of immunomodulatory treatment, including steroids, at least one month prior to blood sampling. Patients were seen at Erasmus Medical Center (*Rotterdam, the Netherlands*) and clinical information on all subjects was extracted from institutional patient records (Table S1).

Isolation of mononuclear cells from peripheral blood

As described previously,[1] peripheral blood mononuclear cells (PBMCs) were isolated from blood either by density gradient centrifugation using Ficoll-Paque™ Plus (*GE Healthcare*) or with vacutainer® CPT™ tubes (*BD Biosciences*). PBMCs were either freshly used or frozen in liquid nitrogen for later use.

In vitro differentiation assay

Total $CD27^+$ memory B cells were sorted from thawed blood by fluorescence activated cell sorting (FACS) using fluorochrome-labeled monoclonal anti-human antibodies (Table S2) on the FACSAria™ Fusion cell sorter (*BD Biosciences*) and the FACSAriaIII sorting machine (*BD Biosciences*). As reported previously,[1] cells were co-cultured with irradiated 3T3-CD40L fibroblasts and stimulated with IL-21 (*Thermo Fisher Scientific*, 50 ng/mL) and IFN-γ (*Peprotech*, 50 ng/mL). After incubation for 6 days (37°C, 5% CO_2), cells were analyzed by spectral flow cytometry on a 5-laser Cytek Aurora flow cytometer (*Cytek Biosciences*).

Flow cytometry

PBMCs or *in vitro*-cultured memory B cells were stained with the Zombie NIR™ Fixable Viability Kit (*Biolegend*) for 10 min. at RT in the dark or with eBioscience™ Fixable Viability Dye eFluor™ 450 (*Thermo Fisher Scientific*) for 15 min. at 4°C in the dark. Subsequently, cells were washed and stained extracellularly in Brilliant Stain Buffer (BSB) or FACS buffer for 15-20 min. at RT in the dark or for 30 min. at 4°C in the dark using fluorochrome-labeled monoclonal anti-human antibodies (Table S2). Afterwards, cells were fixed and permeabilized using the eBioscience™ Foxp3 Transcription Factor Staining Buffer Set (*Invitrogen*) according to manufacturer's protocol. Then, cells were washed and stained

intracellularly in Perm buffer at 4°C in the dark using fluorochrome-labeled monoclonal anti-human antibodies (Table S2). Cells were measured with a 5-laser Cytek Aurora flow cytometer (*Cytek Biosciences*) or a LSRII-Fortessa flow cytometer (*BD Biosciences*). Data analysis was done using OMIQ software from Dotmatic (www.omiq.ai, www.dotmatics.com).

Statistics

All statistical analyses were conducted using GraphPad Prism 9 (Graphpad Software). Grubbs' tests, Mann-Whitney U tests, Kruskal-Wallis tests followed by Dunn's post-hoc analysis, and Spearman correlation coefficients were applied as appropriate. Statistical significance ($p < 0.05$; two-tailed) is indicated in all graphs by asterisks. All data are shown using the mean ± standard error of the mean (SEM).

Supplementary references

1. van Langelaar, J. *et al.* Induction of brain-infiltrating T-bet–expressing B cells in multiple sclerosis. *Annals of Neurology* **86**, no. 2 (2019 Aug): 264–278.

Supplementary Tables and Figures

Table S1: Summarized clinical information of donors included in this study

Cohorts	Subject, n	Female, n (%)	Age in years, median (IQR)[1]	Relapsing onset	Progressive disability[1]	Disease duration in years, median (IQR)[1]
Thawed blood samples:						
• Healthy	12	9 (75%)	42.5 (29.75-59.25)	-	-	-
- young	6	3 (50%)	29.5 (28.0-31.0)	-	-	-
- old	6	6 (100%)	59.5 (56.0-64.0)	-	-	-
• MS	12	6 (50%)	46.5 (38.5-57.25)	-	-	10.5 (4.5-15.5)
- relapsing	6	3 (50%)	38.0 (34.0-44.0)	6 (100%)	0 (0%)	3.5 (1.0-8.0)
- progressive	6	3 (50%)	54.5 (47.0-58.0)	6 (100%)	6 (100%)	13.5 (10.0-21.0)
• AE	10	6 (60%)	50.5 (47.0-62.0)	-	-	1.5 (<1-13.0)
- anti-LGI1	4	3 (75%)	55.5 (50.5-66.5)	-	-	<1 (<1-<1)
- anti-IgLON5	3	0 (0%)	62.0 (47.0-74.0)	-	-	2.0 (1.0-3.0)
- anti-GAD65	3	3 (100%)	43.0 (40.0-48.0)	-	-	17.0 (13.0-25.0)
• RDD	6	3 (50%)	39.5 (26.0-49.0)	-	-	1.0 (<1-3.0)
- anti-AQP4 NMOSD	3	2 (67%)	46.0 (26.0-58.0)	-	-	<1 (<1-3.0)
- MOGAD	3	1 (33%)	33.0 (20.0-49.0)	-	-	1.0 (1.0-9.0)
Fresh blood samples:						
• MS (relapsing)	25	15 (60%)	38.0 (33.5-46.5)	25 (100%)	0 (100%)	-
• MS (progressive)	33	19 (58%)	60.0 (51.5-65.0)	0 (0%)	33 (100%)	-

[1] at the moment of sampling

Table S2: Fluorochrome-labelled monoclonal anti-human antibodies used for flow cytometry or FACS

Antibody marker	Fluorochrome	Clone	RRID	Catalogue number	Company
***Ex vivo* immune cell phenotyping**					
CD3	AF532	UCHT1	AB_11218675	58-0038-42	Thermo Fisher Scientific[1]
CD4	cFluor YG584	SK3	-	SKU R7-20042	Cytek Biosciences[2]
CD8	cFluor V547	SK1	-	SKU R7-20064	Cytek Biosciences
CD11c	BV711	B-ly6	AB_2738019	563130	BD Biosciences[3]
CD16	NovaFluor B 610-70S	3G8	AB_3098058	H006T03B06-A	Thermo Fisher Scientific
CD20	AF700	2H7	AB_1727447	560631	BD Biosciences
CD21	BUV661	B-ly4	AB_2871013	741605	BD Biosciences
CD27	PE-Cy5	O323	AB_10717249	15-0279-42	Thermo Fisher Scientific
CD38	APC-Fire810	HIT2	AB_2860783	303550	Biolegend[4]
CD45RA	BUV496	5H9	AB_2870749	741182	BD Biosciences
CD56	BB700	B159	AB_2722504	566400	BD Biosciences
CXCR3	PE-Fire810	G025H7	AB_2894484	353760	Biolegend
IgD	Pacific Blue	IA6-2	AB_2561596	348224	Biolegend
IgM	BV570	MHM-88	AB_10913816	314518	Biolegend
IgG	BV786	G18-145	AB_2738684	564230	BD Biosciences
IgA	FITC	IS11-8E10	-	130-113-475	Miltenyi Biotec[5]
T-bet[6]	PE-Cy7	4B10	AB_2561760	644824	Biolegend
***In vitro* and *ex vivo* ASC phenotyping**					
CD3	AF700	SK7	AB_2563419	344822	Biolegend
CD19	BV785	HIB19	AB_2563442	302240	Biolegend
CD27	BV421	M-T271	AB_11153497	562513	BD Biosciences
CD38	PerCP-Cy5.5	HIT2	AB_893314	303522	Biolegend
**Fluorescence activated cell sorting (FACS)**					
CD3	AF700	SK7	AB_2563419	344822	Biolegend
CD19	BV785	HIB19	AB_2563442	302240	Biolegend
CD27	BV421	M-T271	AB_11153497	562513	BD Biosciences
CD38	APC	HIT2	AB_314361	303510	Biolegend
Apotracker green	FITC	-	-	427403	Biolegend

[1]Thermo Fisher Scientific, Landsmeer, The Netherlands [2]Cytek Biosciences, Amsterdam, The Netherlands [3]BD Biosciences, Erembodegem, Belgium [4]Biolegend, London, United Kingdom [5]Miltenyi Biotec, Leiden, the Netherlands [6]Intracellular staining

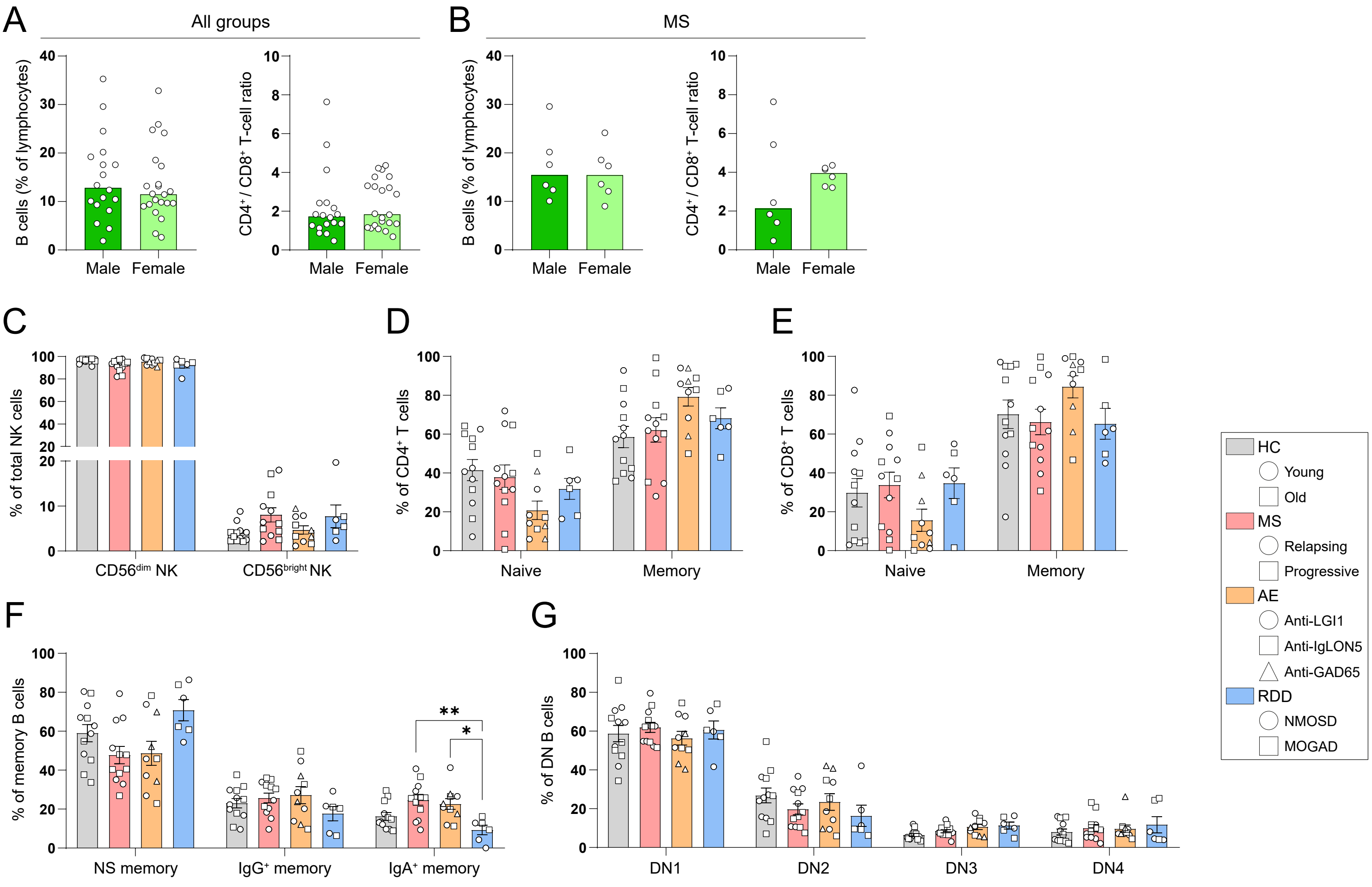

Figure S1: No major differences in circulating B-, T- and NK-cell subsets between MS and other CNS AIDs. B-cell frequencies and $CD4^+$/$CD8^+$ T-cell ratios stratified by sex in both the total study population (A) and the MS group (B). (C) $CD56^{dim}$ and $CD56^{bright}$ NK-cell frequencies within total NK cells. (D) Naive and memory frequencies within $CD4^+$ T cells. (E) Naive and memory frequencies within $CD8^+$ T cells. (F) Non-switched (NS) and class-switched (CS; IgG^+ or IgA^+) frequencies within memory B cells. (G) Relative distribution of double negative (DN; $CD27^-IgD^-$) subsets: DN1, DN2, DN3 and DN4. All data are presented as mean ±SEM and analyzed using Mann-Whitney U tests (A-B) or Kruskal-Wallis and Dunn's post-hoc tests (C-G). Outliers were identified and excluded using Grubbs' test. *p<0.05, **p<0.01.

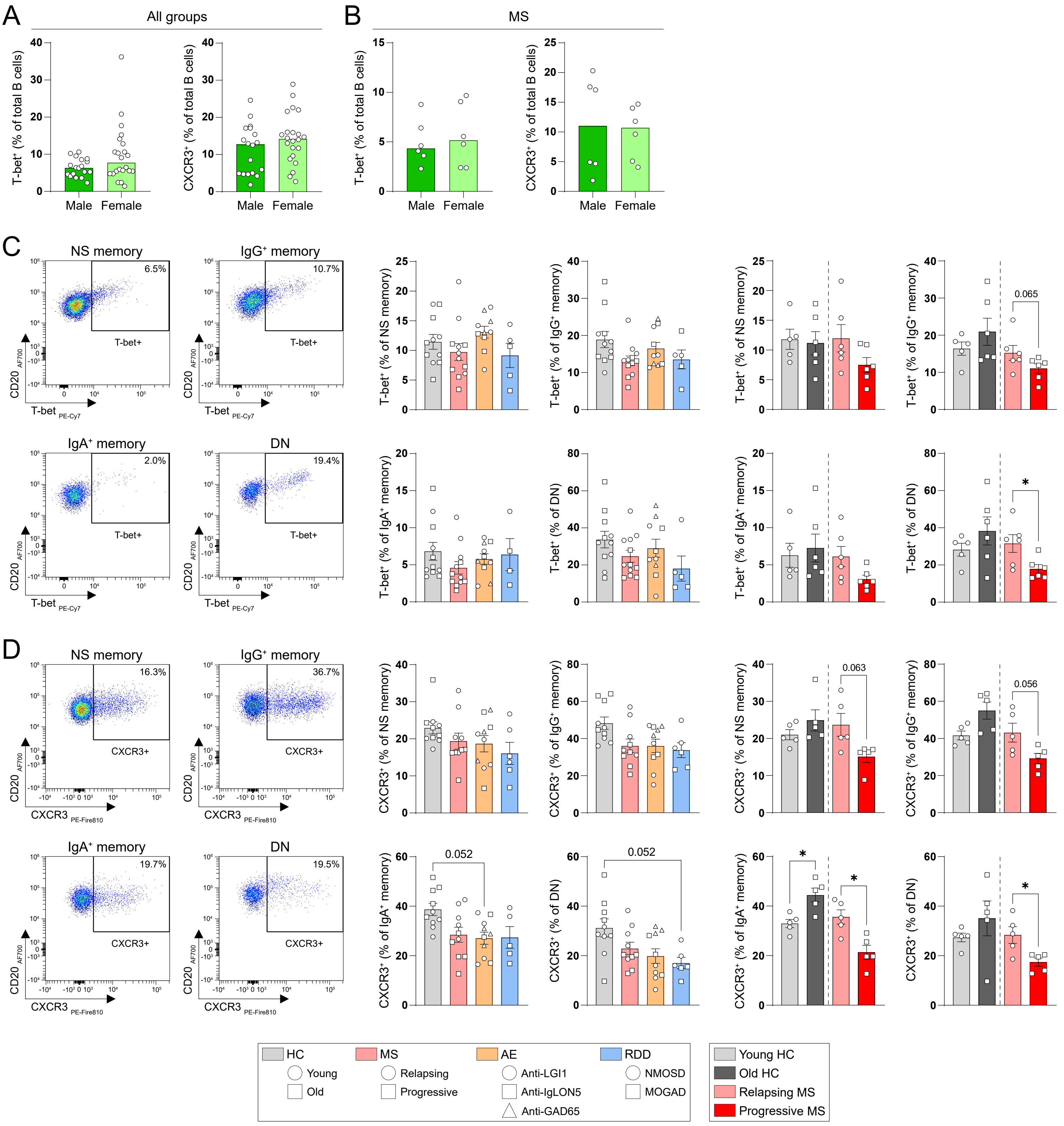

Figure S2: Frequencies of circulating T-bet+ and CXCR3+ B cells stratified by sex and within different memory subsets. T-bet+ and CXCR3+ fractions within B cells in males versus females in both the total study population (A) and the MS group (B). (C-D) Representative gating and frequencies of T-bet+ and CXCR3+ fractions within non-switched (NS) memory, IgG+ memory, IgA+ memory, and double negative (DN; CD27-IgD-) B cells. All data are presented as mean ±SEM and analyzed using Mann-Whitney U tests (A-D) or Kruskal-Wallis and Dunn's post-hoc tests (C-D). Outliers were identified and excluded using Grubbs' test. *p<0.05.

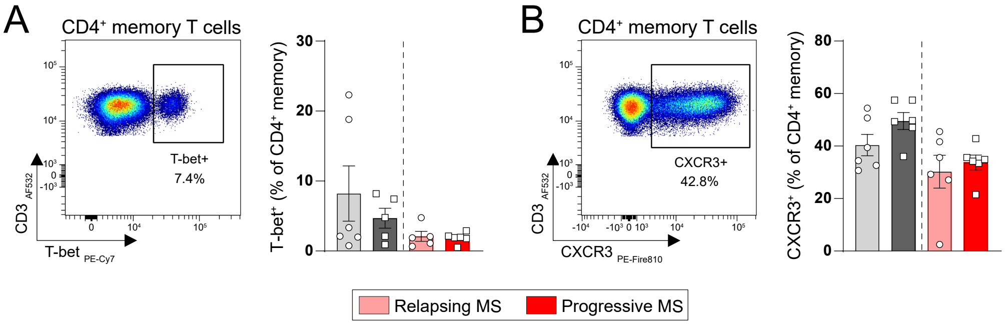

Figure S3: Comparable T-bet+ and CXCR3+ proportions within circulating CD4+ memory T cells from people with relapsing and progressive MS. (A-B) Representative gating and frequencies of T-bet+ and CXCR3+ cells within CD4+ memory T cells from blood of people with relapsing and progressive MS. All data are presented as mean ±SEM and analyzed using Mann-Whitney U tests. Outliers were identified and excluded using Grubbs' test.

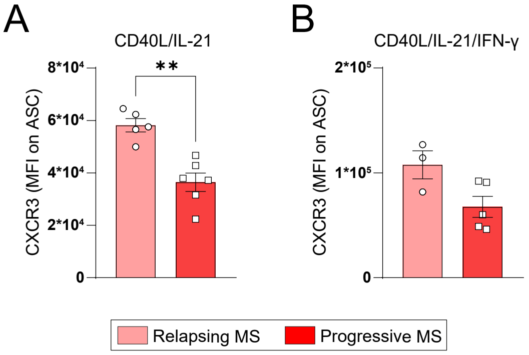

Figure S4: Reduced CXCR3 expression on in vitro-induced ASCs from people with progressive versus relapsing MS. (A-B) CXCR3 expression on ASCs induced from memory B cells that were co-cultured in vitro for 6 days with 3T3-CD40L fibroblasts, IL-21 and/or IFN-γ. All data are presented as mean ±SEM and analyzed using Mann-Whitney U tests. Outliers were identified and excluded using Grubbs' test. **$p<0.01$.
